# The Role of miR-29s in Human Cancers—An Update

**DOI:** 10.3390/biomedicines10092121

**Published:** 2022-08-29

**Authors:** Thuy T. P. Nguyen, Kamrul Hassan Suman, Thong Ba Nguyen, Ha Thi Nguyen, Duy Ngoc Do

**Affiliations:** 1 Division of Radiation and Genome Stability, Department of Radiation Oncology, Dana-Farber Cancer Institute, Boston, MA 02215, USA; 2 Department of Fisheries, Ministry of Fisheries and Livestock, Dhaka 1205, Bangladesh; 3 Department of Anatomy, Biochemistry, and Physiology, John A. Burns School of Medicine, University of Hawaii at Manoa, Honolulu, HI 96813, USA; 4 Institute of Research and Development, Duy Tan University, Danang 550000, Vietnam; 5 Center for Molecular Biology, College of Medicine and Pharmacy, Duy Tan University, Danang 550000, Vietnam; 6 Department of Animal Science and Aquaculture, Dalhousie University, Truro, NS B2N 5E3, Canada

**Keywords:** cancer, microRNA, miR-29, biomarker, therapeutic target

## Abstract

MicroRNAs (miRNAs) are small non-coding RNAs that directly bind to the 3’ untranslated region (3’-UTR) of the target mRNAs to inhibit their expression. The miRNA-29s (miR-29s) are suggested to be either tumor suppressors or oncogenic miRNAs that are strongly dysregulated in various types of cancer. Their dysregulation alters the expression of their target genes, thereby exerting influence on different cellular pathways including cell proliferation, apoptosis, migration, and invasion, thereby contributing to carcinogenesis. In the present review, we aimed to provide an overview of the current knowledge on the miR-29s biological network and its functions in cancer, as well as its current and potential applications as a diagnostic and prognostic biomarker and/or a therapeutic target in major types of human cancer.

## 1. Introduction

MicroRNAs (miRNAs) are a class of small non-coding RNAs (ncRNAs) with approximately 18–24 nucleotides in length. The miRNAs control gene expression post-transcriptionally by binding to the 3′-untranslated region (3′-UTR) of its targeted messenger RNAs (mRNAs), resulting in mRNA cleavage or translation repression [1,2]. Noticeably, miRNAs have been indicated to be involved in all cancer stages from tumor initiation to metastasis [3], suggesting that miRNA could potentially be used as a diagnostic and/or prognostic biomarker or a therapeutic target in cancer treatments [4]. Among dozens of miRNAs that are abnormally expressed in cancer, miR-29s have been recognized as the critical one that acts as both oncogenic and tumor-suppressor regulators in cancer [5].

**Figure 1:**
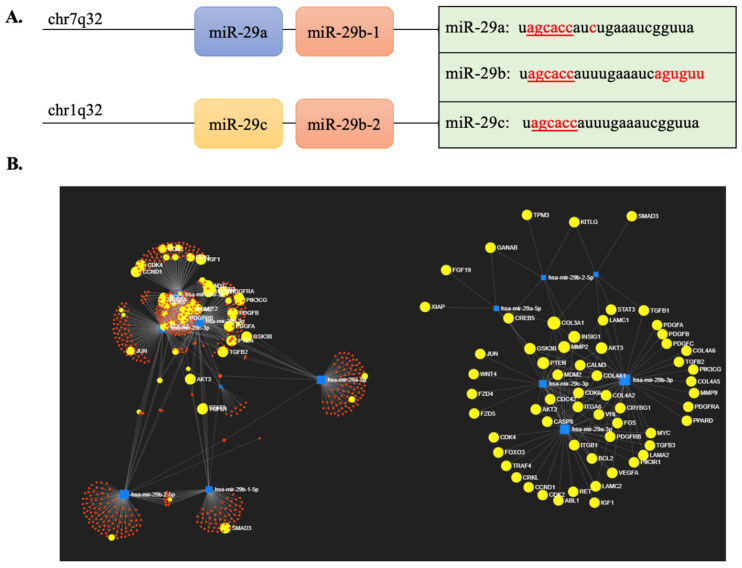
The miR-29 family members and their potential targets. (**A**) Mature sequences and chromosomal locations of miR-29s. MiR-29a/miR-29b-1 cluster is located in chr7q32, whereas miR-29b-2/miR-29c is in chr1q32. The mature miR-29s share a common seed region (agcacc) but are different at the cytosine position in miR-29a and a nucleotide sequence at the end of miR-29b (aguguu) which is known for nuclear localization. (**B**) The potential targets of miR-29s. The visualization is based on miR-29s’ experimental targets of the miRNet platform (https://www.mirnet.ca, accessed on 24 July 2022). Each dot represents a gene. The yellow dots indicate the target genes involved in cancer pathways.

The miR-29 family consists of two clusters namely miR-29a/miR-29b-1 and miR-29c/miR-29b-2 located on chromosome 7q32.3 and 1q32.2, respectively [6]. Mature miR-29s in humans, mice, and rats share a common seed region [7] that plays a role in determining their target mRNAs. However, miR-29s exhibit differential expression and regulation in various cases. The miR-29a is the most abundantly expressed at all stages of the cell cycle; whereas, miR-29b exhibits low-level expression, rapid degradation, and becomes stable during mitosis; and miR-29c is undetectable [8]. Pulse-chase experiments have indicated that the miR-29a mimic has greater stability compared to miR-29b in Hela cells [9]. Besides, a deep sequencing miRNAs analysis revealed that miR-29s had distinct subcellular distributions [10]. While miR-29a is more prevalent in the cytoplasm, miR-29b is mostly localized in the nucleus [10]. The nuclear localization of miR-29b is mostly due to six nucleotides (nts) localized at the end of its sequences [8] (Figure 1A). Additionally, miR-29b-specific knock-out disrupts the tertiary structure of miRNA clusters and changes the sequence or structure of promoters, resulting in lower expression of miR-29a and miR-29c [11]. These results indicated that miR-29s may function differently in different conditions in both corporative and separate manner.

The miR-29s play an important role in a multitude of pathophysiological processes. According to the miRNet database, 677 human genes have been identified as potential targets of the hsa-miR-29 family (https://www.mirnet.ca; accessed on 27 November 2021), and many of them are involved in cancer pathways (Figure 1B). Several studies have revealed a strong antifibrotic property of miR-29s in multiple organs, such as heart [12], liver [13], lung [14], and kidney [15]. Specifically, the miR-29s negatively regulated multiple extracellular matrix (ECM) proteins [16,17,18], which are essential for matrix deposition, epithelial-mesenchymal transition (EMT) [14], and the progression of fibrosis. Additionally, dysregulated miR-29s were also identified in various conditions in liver fibrosis [13,19,20], cardiovascular diseases [12,21], and hepatitis C virus infection [13,19,22]. Intravenous injection of miR-29 mimics may reduce collagen biosynthesis and reverse pulmonary fibrosis [23].

Moreover, the expression of the miR-29 family was reduced in various types of cancer, suggesting their tumor-suppressing capacity as well as their potential role as a diagnostic and prognostic marker in these cancer types. Several studies have indicated that miR-29s can negatively regulate DNA methyl transferase proteins (DNMT3A/3B) in the lung, gastric, and liver cancer [24,25,26]. Additionally, miR-29s have also been found to suppress the expression of histone deacetylases (HDAC4) [27,28], thymine DNA glycosylase (TDG), and ten-eleven translocation 1 (TET1) [29]. Furthermore, miR-29s could act as pro-apoptotic, anti-proliferative, anti-metastatic/EMT, and immunomodulatory factors by directly binding to 3′-UTR of the target genes such as myeloid cell leukemia 1 (MCL1) [30], cyclin-dependent kinase 6 (CDK6) [31], cell division cycle 42 (CDC42) [32] and matrix metalloproteinase 2 (MMP2) [17] in human cancers. Therefore, miR-29-targeted interventions can be used as a therapeutic approach to inhibit tumorigenesis and invasion in different types of cancer. In contrast, miR-29s were also upregulated in several types of cancer. For example, miR-29a was upregulated in colorectal cancer metastasis [33], breast cancer [34], and in the urine of bladder cancer (BC) patients [35], while miR-29c-5p was significantly increased in the advanced stage of BC serum samples [36]. These studies suggested an oncogenic role of miR-29s and the potential link between the dysregulation of miR-29s and the carcinogenesis in these cancer types.

In the current review, we summarize and discuss the function of miR-29s across human cancers and the use of miR-29s as diagnostic, prognostic, and therapeutic biomarkers.

## 2. miR-29 Functions in Cancers

**Figure 2:**
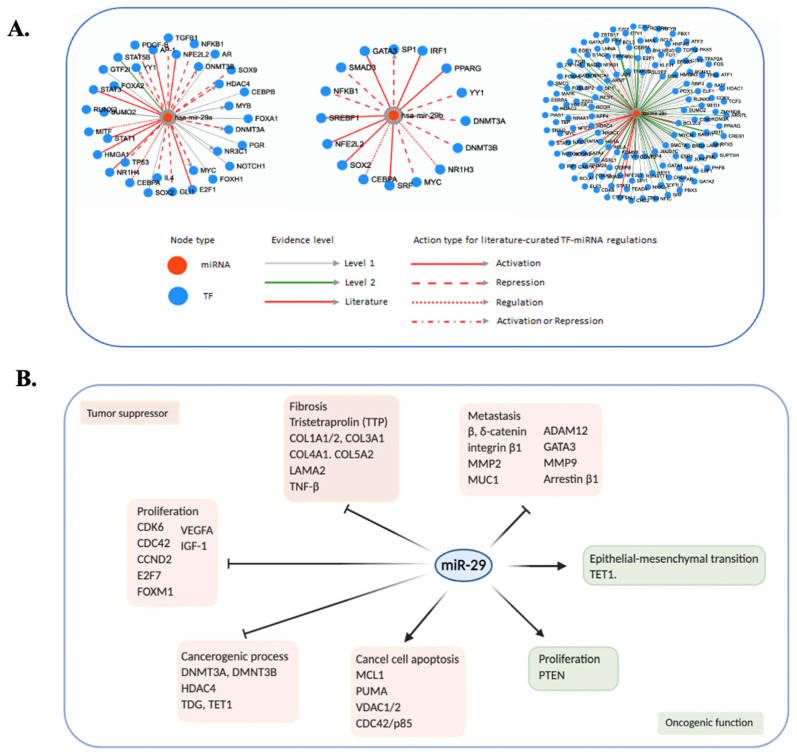
Roles of miR-29s in human cancers. (**A**) MiR-29s modulated different transcription factors. The visualization is based on miR-29s in transcriptome networks of the TransmiR v2.0 platform (cuilab/cn/transmir). The red dots represent has-mir-29a, -29b, and -29c from left to right, respectively, while the blue dots represent different cancer-related transcription factors. (**B**) cancer-related miR-29s targets. MiR-29s can act as both tumor suppressor and tumor inducer genes by contributing to different cancer pathways such as cancer cell apoptosis, cancerogenic process, proliferation, fibrosis, and metastasis.

The miR-29s play a central role in the transcriptome networks, in which miR-29c was the most frequently reported one for its function in regulation of transcription factors. It has been reported that all three miR-29s were regulated by c-Myc, Yin and Yang 1 (YY1), and CCAAT/enhancer-binding protein-α (CEBPA). Particularly, c-Myc suppressed miR-29s transcription through a co-repressor complex with histone deacetylase 3 (HDAC3) and Enhancer of zeste homolog 2 (EZH2); and combined inhibition of HDAC3 and EZH2 restored miR-29s expression levels, which, in turn, caused lymphoma growth suppression [37]. Nuclear factor kappa B (NF-kB)-activated YY1 also inhibited miR-29s expression in myogenesis and rhabdomyosarcoma [38]. The CBEPA, on the other hand, selectively induced the transcription of miR-29a/b-1, but not miR-29b-2/c [39]. In addition, miR-29s have also been reported to be involved in the modulation of a set of transcription factors (Figure 2A), including tumor suppressors and oncogenic genes that are involved in different cancer biological pathways (Figure 2B).

The DNA methylation is a well-studied epigenetic gene silencing mechanism in mammalian cells and organisms [40]. Promoter hypermethylation of a tumor suppressor gene causes gene inactivation and thus may lead to cancer development. Numerous tumor suppressor genes were hypermethylated in various human cancers, such as BRCA1 in early breast cancer, MLH1 (mutL homolog (1) gene in colorectal cancer (CRC), and VHL (von Hippel–Lindau) gene in renal cell cancer [41]. The miR-29s directly suppress DNA methyltransferase enzymes and two other DNA methylation proteins, Thymine DNA Glycosylase (TDG) and Tet Methylcytosine Dioxygenase (TET1) [29,42]. According to Morita and colleagues, miR-29s protect cells from tumorigenesis by maintaining the existing DNA methylation profiles. In lung cancer, miR-29s induced silencing of DNMT3A/3B by binding to their 3′-UTR, thereby promoting tumor growth [24]. In multiple myeloma, miR-29b mimics reduced HDAC4 expression and myeloma cell migration, while increasing histone H4 acetylation and apoptosis [28].

Cyclin-dependent kinases (CDKs) were known for their central roles in cell cycle regulation. The CDK6 complexes promoted cancer cells to enter the S phase, thereby enhancing cell proliferation and growth. Numerous studies have reported that miR-29s suppressed the proliferation and invasion of cancer cells by inhibiting the expression of CDK6 in different types of malignancies, including osteosarcoma [31], and gastric carcinoma [43,44], and bladder cancer [45]. Additionally, miR-29s have also been shown to arrest the cell cycle at G0/G1 phases in gliomas [32] and breast cancer [46], and the G1-S phase in acute myeloid leukemia (AML) [47] by targeting cell division cycle 42 (CDC42) and cyclin D2 (CCND2), respectively; or to suppress tumor growth by repressing angiogenesis genes such as vascular endothelial growth factor (VEGF) [48,49] and insulin-like growth factor *1* (IGF-1) [50] in osteosarcoma and gastric cancer cells.

The dysregulation of ECM remodeling proteins is a high-risk factor for cancer. Fibrosis is a complex process involved in the deposition and reorganization of the matrix, leading to the EMT and thus metastasis of cancer cells. Transforming growth factor beta (TGF-β) receptor binding induced the phosphorylation of the downstream transcription factors SMAD2/3 to stimulate fibrogenic gene expression, including COL1A1, COL1A2, and COL3A1 [51]. The miR-29s have been reported to be significantly downregulated by TGF-β/SMAD signaling in renal fibrosis [15,52]. Overexpression of miR-29s, however, can inhibit the expression of TGF-β1 and SMAD through a feedback loop, thus protecting cells from fibrosis development [53]. Besides, miR-29s also have negatively regulated other ECM-related genes such as laminins, integrins, MMPs, and ADAMs, strongly indicating its anti-fibrotic activity [16,17,54,55].

The miR-29s have also been linked to cancer metastasis, an indicator of poor prognosis. It has been reported that the expression of miR-29s were induced in chemo drugs-treated gastric cancer and that increased expression of miR-29c suppressed gastric cancer cell migration and invasion by negatively regulated *δ*-catenin [56]. Similarly, a strong downregulation of miR-29c has also been observed in pancreatic cancer, which was accompanied by hyperactivation of Wingless-related integration site (Wnt) signaling pathways [57]. Overexpression of miR-29c inhibited the Wnt/β-catenin signaling by down-regulating Wnt’s upstream regulators, resulting in reduced invasion and metastasis in pancreatic cancer [57]. Membrane-bound mucin (MUC1), a stabilizer of β-catenin and Wnt/β-catenin signaling, has also been identified to be inhibited by miR-29a [58]. Another group of metastasis-induced proteins is the MMP family which has also been reported as a direct target of miR-29s. Particularly, miR-29b negatively regulated MMP2/9 by binding to its 3′-UTR, causing cell migration suppression in gastric cancer [17], or osteosarcoma cells [59].

Moreover, miR-29s induced apoptosis in cancer cells by negatively regulating anti-apoptotic proteins such as MCL-1 [30], VDAC1/2 [60], and CDC42/p85 complex [61]. Specifically, miR-29s elevated p53 levels and promoted the p53-dependent apoptotic pathway by directly suppressing two p53 inhibitors, p85 alpha and CDC42 [61]. Besides, by inhibiting the expression of MCL-1, an anti-apoptotic protein, and VDAC1/2 that is essential for the release of cytochrome C from mitochondria to the cytoplasm, miR-29a promoted apoptosis in cancer cells [30,60].

Lastly, some other studies have reported a contradictory role of miR-29s, which functioned as an oncogene in several types of human cancers. In osteosarcoma, for instance, miR-29s were shown to be an oncogenic factor where their downregulation resulted in significantly reduced cell growth and colony formation of osteosarcoma MG-63 cells, probably via miR-29/TGF-β1/PUMA (p53 upregulated modulator of apoptosis) axis [62]. Knockdown of PUMA in these cells, however, reversed miR-29s-induced cell growth suppression and apoptosis [62], due to its ability to induce mitochondrial translocation of Bax (Bcl-2 Associated X-protein) [63]. In addition, miR-29a was upregulated in estrogen receptor-negative (ER^-^) breast cancer that was strongly associated with tumor metastasis and shorter OS (overall survival) in patients with breast cancer [62]. The MiR-29a was proposed as a tumor activator that induces cell proliferation and migration by targeting and inhibiting TET1 [64]. Moreover, overexpression of miR-29s caused a reduction of Phosphatase and Tensin-Like Protein (PTEN), a tumor suppressor, resulting in a restoration of proliferation and migration in osteosarcoma cells [65]. In some cases, the functions of miR-29s have not been identified [66], suggesting that miR-29s can function as either an oncogene or a tumor suppressor depending on specific cellular contexts.

## 3. MiR-29s as Biomarkers

The miR-29s have been repeatedly reported for their abnormal expression across human cancers, suggesting their roles in cancer initiation and progression as well as their potential to be used as diagnostic and/or prognostic biomarkers in cancers. In this part, we summarized the potential use of miR-29s as biomarkers in major types of human cancer.

### 3.1. MiR-29s as Biomarkers in Colorectal Cancer

**Figure 3:**
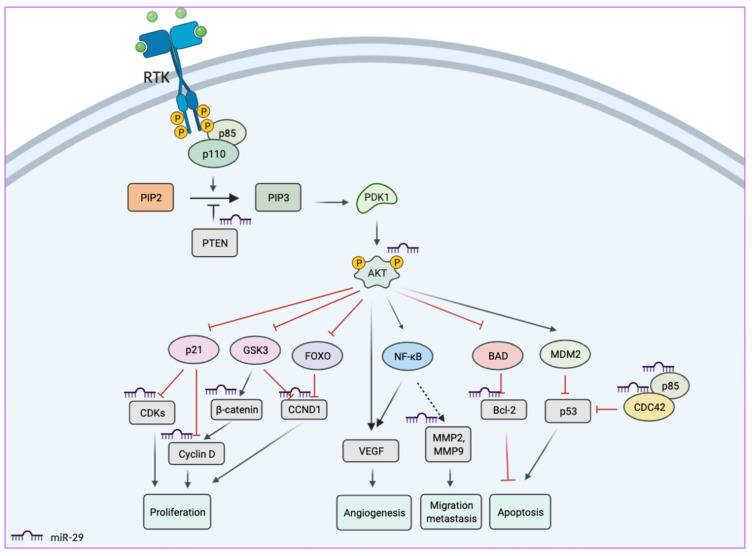
Potential functions of miR-29s in PI3K/AKT signaling pathways. MiR-29s directly inhibit the transcription of various PI3K/AKT downstream factors, resulting in the suppression of cell proliferation, angiogenesis, cell migration, and metastasis, while causing induction of cancer cell apoptosis.

Integrative bioinformatics analysis has revealed the biological functions of the miR-29 family in CRC (colorectal cancer) occurrence and development [67]. Accordingly, pathway enrichment analysis indicated that the miR-29s-targeted genes were associated with the PI3K-AKT signaling pathway, p53-mediated apoptosis, cell cycle, FOXO (forkhead box transcription factors) signaling pathway, and miRNAs in cancer (Figure 3). Thus, miR-29s have been previously proposed to be used as potential biomarkers for CRC diagnosis and prognosis [68,69,70,71,72]. The testing samples ranged from serum, plasma, feces, and tissues were used to measure the levels of miR-29s in CRC. For quantitative measurement of the diagnosis accuracy, each study has calculated the area under the curve (AUC) of summary receiver operating characteristic (ROC), sensitivity, and specificity, which are listed in Table 1. Recently, a systemic meta-analysis based on a hundred single studies revealed that it was valuable to use miR-29s expression alone or in combination with other biomarkers to diagnose or prognoses CRC. Using the miR-29s alone method, however, had lower accuracy than combination methods, with AUC, sensitivity, and specificity of 0.82, 70%, 81%, and 0.86, 78%, and 91%, respectively [67]. The expression of miR-29s was mostly downregulated in CRC in all stages and higher in CRC patients with metastasis as compared to those without. In addition, CRC patients with higher miR-29s expression levels exhibited to have better survival outcomes with lower recurrence and metastasis rates [67]. Together, these results suggested the significant role of miR-29s as diagnostic and prognostic biomarkers in CRC.

### 3.2. miR-29s as Biomarkers in Bladder Cancer

Several studies have suggested that urinary miRNAs could be used as potential biomarkers for the noninvasive diagnosis of BC [35,36,76]. Noticeably, the expression of the miR-29 family members was largely varied in BC. For example, miR-29c was significantly increased in the advanced stage of BC serum samples [36]; and miR-29a was up-regulated in urine samples of BC patients [35]. Additionally, after tumor removal, the level of miR-29a in urine samples was significantly decreased, suggesting a correlation between the level of miR-29a in urine and bladder tumor status [35]. Similarly, another study revealed that miR-29b-1 and miR-29c were upregulated in BC T24 cells as compared to normal cells; and knockdown of one of the two miR-29b-1/-29c caused growth suppression in T24 cells [77]. These data indicated the oncogenic role of miR-29s in this type of cancer.

The miR-29c, however, was significantly downregulated in BC samples [45,78,79,80]. Overexpression of miR-29c caused inhibition of cell growth, cell cycle, and cell mobility while induction of apoptosis in T24 cells [45,78]. Additionally, BC cells exposed to exosome-derived miR-29c are more likely to undergo apoptosis, which is achieved by inhibiting BCL-2 and MCL-1 [81]. These contradictory investigations on the roles of miR-29s in BC suggested their importance in biological pathways and their potential to be used as biomarkers for this type of cancer (Table 2). However, identifying whether they function as tumor suppressors or oncogenes in a typical condition of BC is necessary for better understanding their mechanism of action as well as their future applications in prognosis and diagnosis.

### 3.3. miR-29s as Biomarkers in Hepatocellular Carcinoma

Hepatocellular carcinoma (HCC) is the most common cancer in the liver, with a high incidence and mortality rate [82]. The treatment strategy for HCC patients commonly depends on the tumor stage, but curative options are only available for patients with early stages of HCC [83]. Due to the limitation in early diagnosis, one-third of HCC patients cannot receive the appropriate therapy, and another one-third of those experience therapeutic delay, leading to significantly lower OS in HCC patients [84]. This fact suggests an urgent need for novel biomarkers for early and effective diagnosis and prognosis of HCC. As a tumor suppressor, miR-29s have been considered a potential diagnostic and prognostic biomarker for HCC (Table 3). The RNA from different sources such as serum and frozen tissues have been extracted and quantitatively measured by using the qRT-PCR method. Among miR-29 family members, miR-29a exhibited major functions in the liver as well as HCC tissues [85]. The miR-29a was significantly lower expressed in HCC tissues as compared to the controls and overexpression of miR-29a suppressed HCC cell growth by inhibiting the SPARC (Secreted protein acidic, rich in cysteine)-AKT pathway [85]. In hepatocytes, overexpression of miR-29a inhibited PTEN expression, leading to activation of the PI3K/AKT pathway that eventually induced cell migration [86]. The miRNA profile analysis of exosomes isolated from fast- and slow-migrated HCC patient-derived cells (PDCs) revealed a set of differentially expressed miRNAs that were further validated in HCC samples. The results showed a significant downregulation of miR-29b-3p gene in fast-growing PDCs as compared to slow-growing cells, suggesting its role as in metastasis and OS. Consequently, this cluster of miRNAs may serve as a biomarker for the proliferation of HCC cells [87]. Additionally, there is a statistically significant difference in the levels of miR-29c expression in HCC-derived exosomes amongst HCC, hepatitis B virus (HBV) infection, and cirrhosis patients [88]. It was indicated that TLR3 (Toll-like receptor 3) activated macrophages produced exosomes containing miRNA-29s that were proved to be able to prevent hepatitis C virus (HCV) replication in HCC cell line, suggesting the potential use of exosomes comprising miR-29 family members as a therapy to control HCV replication in infected hepatocytes [89]. Additionally, one other study reported that higher expression of miR-29a-3p was associated with a poorer prognosis, shorter OS, and disease-free survival (DFS) in HCC patients [90].

### 3.4. miR-29s as Biomarkers in Pancreatic Cancer

The miR-29a has been validated to be upregulated in tissue samples from patients with pancreatic ductal adenocarcinoma (PDAC) and was considered a potential diagnostic biomarker for this type of cancer [95]. A recent study comparing 38 patients with PDAC and 11 controls revealed that miR-29c-3p was typically downregulated in PDAC as compared to both normal pancreatic tissues and chronic pancreatitis [96]. Similarly, a study that employed high throughput screen figured out 42 candidate miRNAs that were significantly different between pancreatic cancer (PC) and healthy group, and, the miR-29b was noted to be downregulated 2.1 folds in PC samples [97]. Even though the fold-change and significant level of miR-29s were not high enough in both studies, miR-29s were not tested in the validated group and their role as crucial biomarkers in PC has not been confirmed. In one study, Humeau et al. used qRT-PCR to examine the change of 90 miRNAs in PC and identified four significant candidate miRNAs in saliva samples, including miR-29c [98]. However, the sample size (4 controls, 4 pancreatitis, and 7 PC samples) of the study was relatively small, making its finding of miR-29 as a biomarker for PC remains to be further confirmed.

Chemotherapy plays a significant role in the treatment of PC. Gemcitabine (GEM), an inhibitor of DNA synthesis and ribonucleotide reductase, has become a gold standard chemotherapeutic agent for PC [99,100]. Molecularly, miRNA-29a is involved in a PC cell’s response to GEM by regulating the Wnt/β-catenin signaling pathway [101,102]. Wnt3a, an important ligand of the Wnt/β-catenin signaling pathway, has been shown to induce GEM-resistance in PC cells, probably by activating Wnt/β-catenin signaling in these cells [102]. Additionally, miR-29a was detected to be upregulated in PC tissues and cell lines, and its expression level was positively associated with metastasis [103]. Induced expression of miR-29a caused downregulation of tristetraprolin (TTP), thereby elevating the expression of pro-inflammatory factors and EMT markers. Ectopic overexpression of TTP decreased tumor growth and migration in vivo [103].

### 3.5. miR-29s as Diagnostic Biomarkers in Lung Cancer

Histologically, lung cancer is comprised of 85% of non-small-cell lung cancer (NSCLC) and 15% are small-cell lung cancer (SCLC) [104]. Despite recent advances in diagnosis, late diagnosis is still the main reason for poor prognosis and outcomes in lung cancer. It has been noted that miR-29c was overexpressed in the serum of NSCLC patients as compared to the normal controls [105,106]. Similarly, miR-29a was also found to be upregulated in peripheral blood of lung cancer patients as compared to the healthy control individuals [107]. These findings suggested that miR-29a and miR-29c could be used as potential diagnostic biomarkers for lung cancer. Additionally, Liu et al. reported that miR-29a was strongly downregulated in lung cancer tissues as compared to paired normal tissues and that induced expression of miR-29a suppressed cell proliferation and colony formation of lung cancer cells by targeting and negatively regulating the expression of NRAS (neuroblastoma ras viral oncogene homolog) oncogene. The study has also revealed that miR-29a increased the sensitivity of lung cancer cells to cisplatin treatment and that a combination of miR-29a and cisplatin-induced apoptosis in lung cancer cells, suggesting the potential role of miR-29a as a prognostic biomarker for lung cancer [108]. Recently, it was indicated that NSCLC generated exosomes that contain miR-29a. This miRNA can attach to TLRs in immune cells and elicit protumoral inflammation, hence increasing tumor growth and metastasis [109].

Lung adenocarcinoma (LAC) is a highly aggressive tumor though little is known about its underlying molecular mechanisms. Liu et al. discovered that downregulation of miR-29c was strongly correlated with unfavorable prognosis in stage IIIA LAC patients. The MiR-29c suppressed cell growth, migration, and invasion in human LAC cell lines by directly targeting vascular endothelial growth factor A (VEGFA) [110]. Therefore, miR-29c has been concluded as a tumor suppressor and may be considered a promising prognostic and therapeutic biomarker for LAC [110].

### 3.6. miR-29s as Biomarkers in Leukemia and Lymphoma

Serum miRNAs have been suggested as promising biomarkers for diffuse large B cell lymphoma [111]. Notably, miR-29a and miR-142-3p have been identified to be consistently under-expressed in AML and may act cooperatively in granulopoiesis and monopoiesis [112]. Therefore, dual evaluation of miR-29a and miR-142-3p is more effective for the diagnosis of AML. Additionally, downregulation of miR-29c was identified as a signature for chronic lymphocytic leukemia (CLL) [113], which was greatly correlated with disease progression in CLL patients harboring the 17p deletion [113]. Moreover, miR-29a was significantly downregulated in the bone marrow of pediatric AML patients as compared to the normal controls. The low expression of miR-29a was strongly correlated with shorter relapse-free and OS in these patients [114]. The study suggested that downregulation of miR-29a may be used as a prognostic marker in pediatric AML.

### 3.7. miR-29s as Biomarkers in Kidney Cancer

The miR-29s regulate genes that are closely related to the molecular pathogenesis of renal cell carcinoma (RCC). Serpin family H member 1 (SERPINH1), a direct target of miR-29, was noted to be overexpressed in RCC clinical samples and tyrosine kinase inhibitor failure autopsy specimens. Overexpression of SERPINH1 was significantly associated with advanced tumor stage, pathological grade, and poor prognosis, mostly due to its ability to induce cancer cell migration and invasion [115]. In addition, it was supported with evidence that miR-29b acted as an oncomiR and could be a potential prognostic marker for RCC. The miR-29b promoted proliferation and invasion in SN12-PM6 cells, which inhibited cell apoptosis by directly suppressing the expression of kinesin family member 1B, a tumor suppressor gene that induces cell apoptosis. Upregulation of miR-29b in both cell lines and clinical samples was significantly associated with tumor node metastasis and OS of RCC [116]. These studies suggested the clinical roles of miR-29s in RCC and its potential use as prognostic biomarkers in this type of cancer.

### 3.8. miR-29s as Biomarkers in Breast Cancer

Globally, breast cancer is regarded as one of the most diagnosed and deadly cancers, particularly in the case of women [117,118]. However, the prognosis of breast cancer is not satisfactory, and the 5-year survival rate is lower than 25% [119]. All these phenomena urge the discovery of novel biomarkers for the early diagnosis, and proper therapy of breast cancer. The miR-29s have been studied and suggested as a tumor suppressor in breast cancer [117,120,121]. Wu et al. illustrated that miR-29a was significantly downregulated in breast cancer cells, and its overexpression inhibited cancer cell growth which was achieved by repressing the expression of transcription factor B-Myb [117]. Additionally, overexpression of miR-29a resulted in cell cycle arrest at the G0/G1 phase. The findings denoted the partiality of miR-29a which exerts its tumor suppressor role in breast cancer cell lines by cessation of the cell cycle through negative regulation of CDC42 [46]. Later, Shinden and collaborators investigated the clinicopathological significance of miR-29b in breast cancer cases and illustrated that miR-29b acted as a tumor suppressive miRNA [121], suggesting it is a prominent biomarker for recurrence and metastasis in breast cancer patients. Moreover, overexpression of miR-29b-1/a significantly suppressed proliferation of Tamoxifen (TAM)-resistant breast cancer cells, indicating that miR-29b-1/a functions as a tumor suppressor in these cells [122]. Additionally, BRCA1 (Breast Cancer 1) was reported to bind to a specific region of the promoter and regulate the expression of miR-29b-1-5p. The higher significant level of miR-29b-1-5p as a prognostic marker than other widely used biomarkers signified the potential of this miRNA as a biomarker for BRCA1 deficiency and survival in breast cancer [123]. Consequently, miR-29s are significantly elevated in the whole blood, serum, and tissues samples from breast cancer patients (Table 4). 

Furthermore, overexpressed miR-29s were testified both in tumor tissues and serum of breast cancer patients in comparison to that of healthy individuals [34]. Recently, it was reported that GATA binding protein 3 (GATA3), a transcription factor, elevated the miR-29b level in breast cancer whereas the destruction of miR-29b enhanced metastasis and accelerated EMT. Being a tumor-suppressor gene, the damage of GATA3 in breast cancer resulted in a poor prognosis [124]. A recent study revealed that miR-29a abated cell proliferation and promoted apoptosis in the MCF-7 (Michigan Cancer Foundation-7) cell line by negatively controlling NF-kB (nuclear factor-kappa B) and the levels of cyclinD1 and Bcl-2 proteins [125]. Additionally, the other study revealed that overexpression of miR-29a inhibited cell migration and invasion by negatively regulating Robo1 (Roundabout 1) in breast cancer cells, highlighting the significant role of miR-29a in carcinogenesis breast cancer [126]. Moreover, upregulation of miR-29a induced adriamycin resistance in MCF-7 breast cancer cells, possibly by inhibiting the PTEN/AKT/GSK3β pathway [127]. Treatment with progestin reduced migration and invasion in breast cancer cells, via the miR-29/ATP1B1(ATPase Na^+^/K^+^ transporting β1 polypeptide) axis [128].

**Table 4:** miR-29s as biomarkers in breast cancer.

Samples	Sample Size & Methods	Outcome	Results	Ref.
Blood samples	- 54 patients with Luminal A-like breast cancer and 56 healthy controls - qRT-PCR	MiR-29a was significantly down-regulated in the blood of patients with Luminal A-like breast tumors compared to healthy controls.	Combined miR-29a, miR-181a and miR-652 (AUC: 0.80, sensitivity: 77% and specificity: 74%)	[129]
Serum sample	- 76 breast cancer patients and 52 healthy controls. - SdM-qRT-PCR	MiR-2 was significantly higher in breast cancer patients compared to healthy controls.	MiR-29c AUC: 0.724 (95% CI 0.638–0.810)	[46]
Serum	- 20 breast cancer patients and 20 controls - SOLiD Sequencing (qRT-PCR)	MiR-29a was significantly elevated in the serum of breast cancer patients (*p* < 0.05).	MiR-29a was elevated more than 5-folds by SOLiD sequencing.	[130]
Tissue samples	- 15 breast cancer patients and 15 healthy controls - qRT-PCR	MiR-29a was significantly upregulated in breast cancer as compared with their respective healthy controls (*p* < 0.001).	MiR-29a (AUC:0.969, Sensitivity: 93.3%, specificity: 91.1%)	[131]

CI: confidence interval; AUC: area under the curve, qRT-PCR: quantitative real-time PCR.

## 4. Conclusions and Perspectives

The miR-29s are crucial regulators in numerous types of human cancer, which can act as either tumor suppressors or inducers. By regulating multiple target genes, they are indirectly involved in controlling different cellular pathways including cell proliferation, apoptosis, migration and invasion, and chemotherapeutic sensitivity, thereby contributing to cancer progression, metastasis, and drug resistance. The profound dysregulation of miR-29s in numerous types of cancer and their correlation to the patients’ OS and metastasis have strongly signified them as potential diagnostic and prognostic biomarkers for specific types of cancer. However, due to its flexibility, the application of miR-29s as biomarkers and the development of miR-29s-based therapies need to be verified further for each type and stage of cancer specifically.

Fortunately, the recent advances in sequencing technologies (next generation of sequencing and long-read sequencing) and genome editing allows better validation of the target genes of miR-29s as well as an understanding of the roles of miR-29s in each cancer type. In addition, the rapid adoption of exosomes for the miRNA’s delivery could also support the development of miR-29s for miR-29s-based therapies. In summary, exosomes have several desirable characteristics for delivering miRNAs including small sizes (30–200 nm), being able to cross the blood barriers, being specific to the target cells, and being relatively easy to be engineered. Consequently, the delivery by exosomes of miR-29s to unhealthy/abnormal cells will be adapted for a potential therapeutic approach.

## Figures and Tables

**Table 1:** The potential use of miR-29s as biomarkers in colorectal cancer.

Sample	Sample Size	Outcome	Results	Ref.
Venous blood	114 CRC patients (58 patients with and 56 patients without metastasis)	MiR-29a was significantly increased in CRC patients with metastasis than in those without.	AUC: 80.3% Sensitivity: 75% Specificity: 75%	[73]
Serum	55 CRC patients and 55 normal controls	The serum level of miR-29b was lower in CRC as compared to the normal controls and inversely correlated with the advanced tumor stages.	AUC: 87% Sensitivity: 77% Specificity: 75%	[68]
Tissue Plasma	200 CRC patients and 400 normal controls	The level of miR-29b in plasma and tissue was highly correlated and significantly lower in CRC versus the normal controls.	Tissue: AUC: 88.3% Sensitivity: 81.6% Specificity: 84.9% Plasma: AUC: 74.3% Sensitivity: 61.4% Specificity: 72.5%	[69]
Feces	80 CRC patients and 51 normal controls	The level of miR-29a in feces was significantly lower in CRC versus the normal controls.	AUC: 77.7% Sensitivity: 85% Specificity: 61%	[71]
Serum	160 colorectal neoplasms patients and 77 normal controls	The level of miR-29a in serum was significantly lower in colorectal neoplasms	AUC: 74.1%	[70]
Tissues	245 CRC patients (34 stages I, 63 stages II, 104 stage III, and 44 stages IV)	MiR-29b expression was significantly decreased in tumor versus normal tissues	Higher miR-29b is associated with higher 5-year DFS and OS.	[74]
Tissues	110 CRC patients (51 stages I and 59 stages II)	The level of miR-29a was a positive predictive factor for non-recurrence in stage II CRC.	Higher miR-29a is associated with longer DFS. Sensitivity: 67% Specificity: 88%	[75]

CRC: colorectal cancer; AUC: area under the curve; OS: overall survival; DFS: disease-free survival.

**Table 2:** miR-29s as biomarkers in bladder cancer.

Sample	Sample Size & Methods	Outcomes	Results	Ref.
Serum	- 392 BC samples and 100 normal controls - Bioinformatic analysis	MiR-29c was overexpressed in serum samples	MiR-29c was correlated to the advanced stage and OS time in BC patients.	[36]
Urine	- 276 BC samples: 276 normal controls - MiSeq and qRT-PCR	MiR-29a was upregulated in BC patients.	MiR-29a-3p in combination with six other miRNAs was used for the diagnosis of BC. AUC: 92.3% Sensitivity: 82% Specificity: 96%	[35]
Tissue	- 30 BC samples and 30 normal controls - qRT-PCR	MiR-29c was downregulated in BC. MiR-29c inhibited cell proliferation, migration, and cell cycle progression, and induce apoptosis through AKT signaling.	MiR-29c was inversely associated with bladder tumor stages.	[78]
Tissue	- 106 BC samples and 11 normal samples. - Spotted locked nucleic acid-base oligonucleotide microarrays	MiR-29b and miR-29c were downregulated in BC tumors	Higher miR-29c levels were correlated with longer DFS.	[79]
Specimen	- 108 bladder carcinomas and 29 carcinomas invading the bladder - Microarrays	MiR-29c was significantly under-expressed in progressed tumors.	High expression of miR-29c was associated with a better prognosis.	[80]

BC: bladder cancer; qRT-PCR: quantitative real-time PCR; AUC: area under the curve; OS: overall survival; DFS: disease-free survival.

**Table 3:** MiR-29s as biomarkers in hepatocellular carcinoma.

Sample	Sample Size & Methods	Outcome	Results	Refs
Serum	- 58 NAFLD and 34 normal control - qRT-PCR	MiR-29a: lower in NAFLD patient MiR-29c: unchanged MiR-29b: undetectable	For miR-29a: AUC: 0.679 Sensitivity: 60.87% Specificity: 82.35%	[91]
Tissue	- 266 HCC - Taqman Low-Density Arrays qRT-PCR	MiR-29a-5p was associated with early HCC recurrence, resulting in lower OS	AUC: 0.708 Sensitivity: 74.2% Specificity: 68.2%	[92]
Venous blood	- 174 HCC - qRT-PCR	MiR-29a-3p was higher in both early and late stages of HCC	AUC: 0.71 (95%CI = 0.62–0.78)	[90]
Tissue	- 55 HCC and 55 normal control - qRT-PCR	MiR-29a was downregulated in HCC samples MiR-29a targeted SIRT1 and suppressed the HCC cell cycle and proliferation.	Lower miR-29a is associated with higher tumor size, vascular invasion, poor DFS	[93]
Specimen	- 110 HCC - qRT-PCR	MiR-29a was dramatically decreased in HCC tissues	miR-29a targeted to SPARC, downstream of AKT/mTOR to suppress cell growth.	[94]

HCC: hepatocellular carcinoma; NAFLD: Non-alcoholic fatty liver disease; AUC: area under the curve, qRT-PCR: quantitative real-time PCR; OS: overall survival; DFS: disease-free survival.

## Data Availability

No specific data used in the review article. All information has been provided in the manuscript.
